# Impact of supplementation with dihydroxylated vitamin D_3_ on performance parameters and gut health in weaned Iberian piglets under indoor/outdoor conditions

**DOI:** 10.1186/s40813-023-00307-z

**Published:** 2023-06-15

**Authors:** Carmen Álvarez-Delgado, Inés Ruedas-Torres, José M. Sánchez-Carvajal, Feliciano Priego-Capote, Laura Castillo-Peinado, Ángela Galán-Relaño, Pedro J. Moreno, Esperanza Díaz-Bueno, Benito Lozano-Buenestado, Irene M. Rodríguez-Gómez, Librado Carrasco, Francisco J. Pallarés, Jaime Gómez-Laguna

**Affiliations:** 1 Department of Anatomy and Comparative Pathology, Pathology and Immunology Group (UCO-PIG), UIC Zoonosis y Enfermedades Emergentes (ENZOEM), Faculty of Veterinary Medicine, Agrifood Campus of International Excellence (ceiA3), University of Cordoba, Campus of Rabanales, 14071 Cordoba, Spain; 2 Institute of Virology and Immunology (IVI), Bern, Switzerland; 3 Department of Analytical Chemistry, Nanochemistry University Institute (IUNAN), Faculty of Sciences, Agrifood Campus of International Excellence (ceiA3), University of Cordoba, Campus of Rabanales, 14071 Córdoba, Spain; 4 Department of Animal Health, UIC Zoonosis y Enfermedades Emergentes (ENZOEM), Faculty of Veterinary Medicine, Agrifood Campus of International Excellence (ceiA3), University of Cordoba, Campus of Rabanales, 14071 Cordoba, Spain; 5 COVAP, 14400 Pozoblanco, Cordoba, Spain

**Keywords:** 1,25 dihydroxyvitamin D, Feeding, Pigs, Growth performance, Immunomodulation

## Abstract

**Background:**

Vitamin D may improve innate antimicrobial response and the integrity of the intestinal mucosal barrier representing an alternative to antibiotics for improving pig health. Therefore, benefits of dietary supplementation with a product based on vitamin D_3_ metabolite-rich plant extracts were assessed in 252 purebred Iberian piglets for a period of 60 days. The study group received 1,25 dihydroxyvitamin D (1,25(OH)_2_D) (100 ppm) in the conventional feed, which already included vitamin D (2000 IU in the starter and 1000 IU in the adaptation diets, respectively). Average daily gain (ADG), feed conversion ratio (FCR) and coefficient of variation of body weight (CV-BW) were assessed along the study. Blood samples, from 18 animals of the study group and 14 animals of the control group, were collected at selected time points to determine white blood cell count, concentration of vitamin D_3_ and its metabolites, and IgA and IgG in serum. Histopathology, morphometry, and immunohistochemistry (IgA and FoxP3) from small intestine samples were performed on days 30 and 60 of the study from 3 animals per group and time point.

**Results:**

The ADG (493 *vs* 444 g/day) and FCR (2.3 *vs* 3.02) showed an improved performance in the supplemented animals. Moreover, the lower CV-BW indicated a greater homogeneity in the treated batches (13.17 *vs* 26.23%). Furthermore, a mild increase of IgA and in the number of regulatory T cells in the small intestine were observed in treated pigs.

**Conclusions:**

These results highlight the benefits of this supplementation and encourage to develop further studies along other production stages.

## Background

Current restrictions and limitations in the use of antimicrobials and zinc oxide (ZnO) in pig breeding frequently result in worsening of the health status and loss of performance in piglets, becoming a major concern for swine producers and practitioners [1]. In the search for substitutes for these substances, the use of additives, such as vitamin D or its metabolites, represents a good alternative to improve the health, production, and immune status of the animals [2]. The beneficial effects of this vitamin have been widely demonstrated in the literature [3] and more recently during the fight against the current pandemic COVID-19 [4].

Vitamin D can be obtained from the diet or produced by the skin under the influence of sunlight exposure [5, 6]. Different sources of vitamin D are available for animal feeding including vitamin D_3_ (cholecalciferol), 25OHD_3_ (calcifediol) and 1,25(OH)_2_D_3_ (calcitriol). Calcitriol is the physiologically active metabolite and the molecule responsible for most of vitamin D actions [7]. This metabolite is naturally presented in the leaves or extracts of different plants, such as *Solanum glaucophyllum* (white peach), and has demonstrated a high bioavailability in pigs [8].

Significant roles have been attributed to vitamin D, including calcium and phosphorus homeostasis, skeletal muscle development and immune system functionality [3]. Regarding its immune system effects, vitamin D is an important modulator of the innate antimicrobial response and contributes to the integrity of the intestinal mucosal barrier [5]. Furthermore, it also collaborates in adaptive immunity, stimulating the production of specific antibodies and modulating inflammation through the induction of regulatory T lymphocytes (Tregs) and the activation of dendritic cells, a type of antigen-presenting cell [4, 5, 9].

In this sense, different studies have evaluated the impact of vitamin D supplementation on porcine health, showing promising results [9, 12]. Thus, Konowalchuk et al*.* [10] demonstrated the immunomodulatory role of vitamin D in weanling commercial pigs, impacting on cellular immune parameters, such as the number of leucocytes, particularly in the number of lymphocytes and granulocytes. Other studies have shown how vitamin D supplementation mitigates the effect of rotavirus infection in piglets (decrease in body weight gain, feed intake and villus height, among others), enhancing a protective effect against infections and an improvement in the gastrointestinal health [9, 11]. A recent study performed on sows, showed a higher number of total-born piglets in the 1,25(OH)_2_D_3_ supplemented group, compared with the control one [12]. However, to the author’s knowledge, there are no studies that analyze the impact of this vitamin in Iberian pigs, a fatty breed with a high intake capacity and specific nutritional requirements, different from those of lean commercial breeds, making necessary to provide protein and energy adapted diets to this breed. Furthermore, Iberian pigs are reared mainly in outdoor conditions, which involves a direct interaction with sunlight, subsequently with the ability to synthesize vitamin D_3_ [13–15].

The boosting of the immune status of the animals by using alternative products and supplements is of particular interest in those systems where biosecurity and management practices may be compromised, such as extensive or free-range systems [16]. In this way, vitamin D supplementation has been reported to limit the generalization of tuberculosis-associated lesions in wild boar (*Sus scrofa*) and red deer (*Cervus elaphus*) displaying a negative correlation between serum vitamin D concentration and the intensity of lesions produced by mycobacteria belonging to *Mycobacterium tuberculosis* complex (MTBC) [17].

Therefore, having into account the role of vitamin D as nutritional supplement and its impact on the immune system, the aim of the present study was to evaluate the benefits of the supplementation with 1,25(OH)_2_D_3_ from *Solanum glaucophyllum* extracts in performance, immunological and intestinal morphometry parameters in the post-weaning phase of Iberian piglets.

## Results

### Growth performance: body weight, average daily gain, feed conversion ratio and coefficient of variation of body weight

**Fig. 1:**
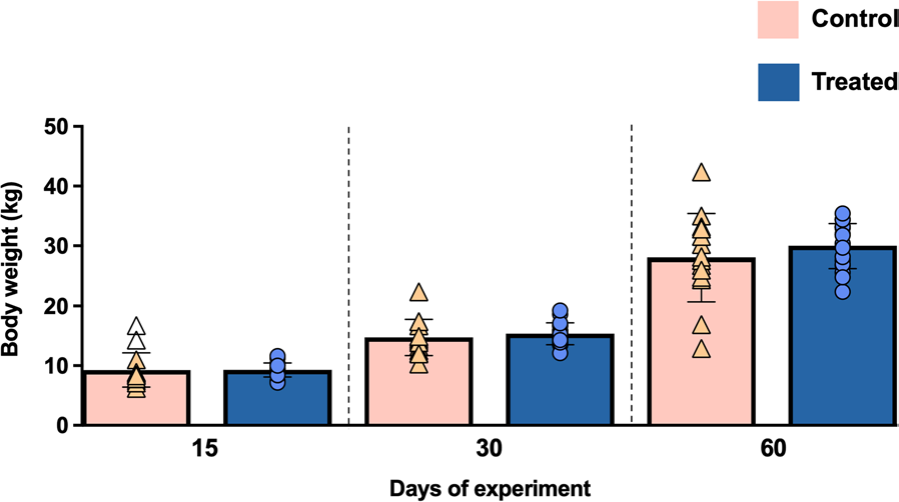
Graphical representation of body weight (kg) along the study. Bars represent the mean of each group at different time points. Orange triangles (control group n = 14) and blue circles (treated group n = 18) represent the individual value per animal within each group. Empty symbols represent outliers

Figure 1 shows the values of body weights of the animals at the different time points of the experimental study. As expected, both groups showed an increase of this parameter throughout the study period, being somewhat higher in the treated group on days 30 and 60 of the experiment, although without significant differences (*P* = 0.278 and *P* = 0.339, respectively)*.* Mean weight values in the control group were 14.73 kg and 28.05 kg on days 30 and 60 of the experiment, respectively, compared to 15.35 kg and 29.99 kg in the treated group at the same time points. Moreover, the Time × Treatment interaction for this variable resulted to be statistically significant (*P* = 0.011; two factors ANOVA).

The coefficient of variation of body weight (CV-BW) from the experimental groups at different time points is shown in Table 1. The treated group showed a lower CV-BW compared to the control one along the study. The most striking differences between both experimental groups were observed at the end of the study (day 60), with only 13.17% of CV-BW in the treated group *vs* 26.23% in the control one (Table 1) (*P* = 0.010, F test).

**Table 1:** Coefficient of variation of body weight (CV-BW, %) in both experimental groups along the study

Group	Date	Coefficient of variation of body weight (CV-BW, %)
Control	1	24.54
Treated	1	21.28
Control	15	26.44
Treated	15	18.98
Control	30	18.58
Treated	30	15.97
Control	60	26.23
Treated	60	13.17^*^

^*^P = 0.010, F test

**Fig. 2:**
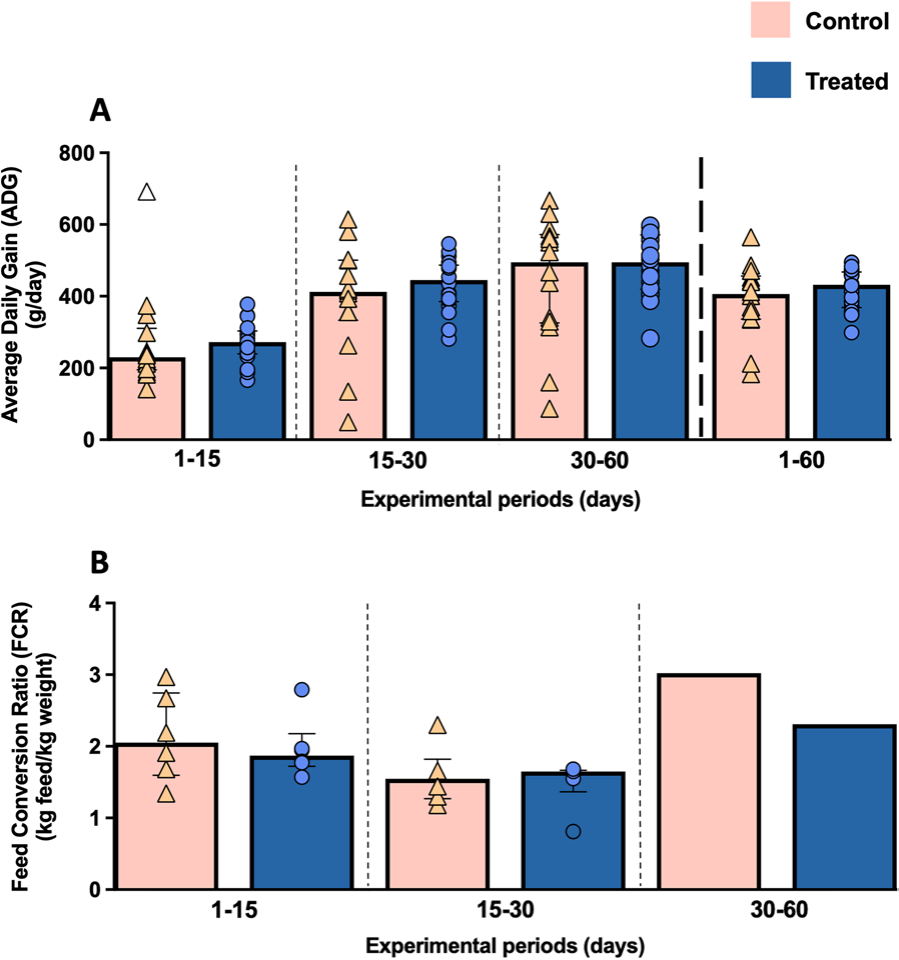
Graphical representations of ADG (**A**) and FCR (**B**) along the study. Bars represent the median of each group at different experimental periods. Orange triangles (control group) and blue circles (treated group) represent the value of each animal (control group n = 14; treated group n = 18) or pen (control group n = 6; treated group n = 6, except in the last study period where only one simple large pen was available for each experimental group) within each group for ADG and FCR, respectively. Empty symbols represent outliers

Results of average daily gain (ADG) are shown in Fig. 2A. Both groups showed a progressive increase in this parameter throughout the study period, but higher values were observed in the treated group in comparison with control group during the 1–15 and 15–30 study periods, although without significant differences (*P* = 0.231 and *P* = 0.299, respectively)*.* Values around 230 g/d (interquartile range, IQR = 115.7) and 412 g/d (IQR = 167.7) were calculated in control group at days 15 and 30 of the experiment, respectively, compared to 272 g/d (IQR = 64) and 445 g/d (IQR = 101.2) in the treated one at the same time points (Fig. 2A). Both groups presented a similar ADG at the end of the study (30–60 study period). The ADG for the entire study period (from day 1 to 60) was also represented, showing a greater increase in the treated group compared to the control one, with values around 432 g/d (IQR = 99.3) and 406.5 g/d (IQR = 120.3), respectively, although without significant differences *(P* = 0.314) (Fig. 2A).

Regarding feed conversion ratio (FCR), a slight decrease was noticed in treated piglets in comparison with control group at the study period 1–15 (FCR 1.87, IQR 0.45, for treated group; and FCR 2.05, IQR 1.15, for control group) (*P* = 0.818) (Fig. 2B). The highest difference between both groups was observed at the end of the study (from day 30 to 60), with a FCR of 3.02 in the control group compared with a FCR of 2.31 in the treated group (Fig. 2B), although since there was only one value per group, statistical significance could not be calculated.

### White blood cell counting

**Fig. 3:**
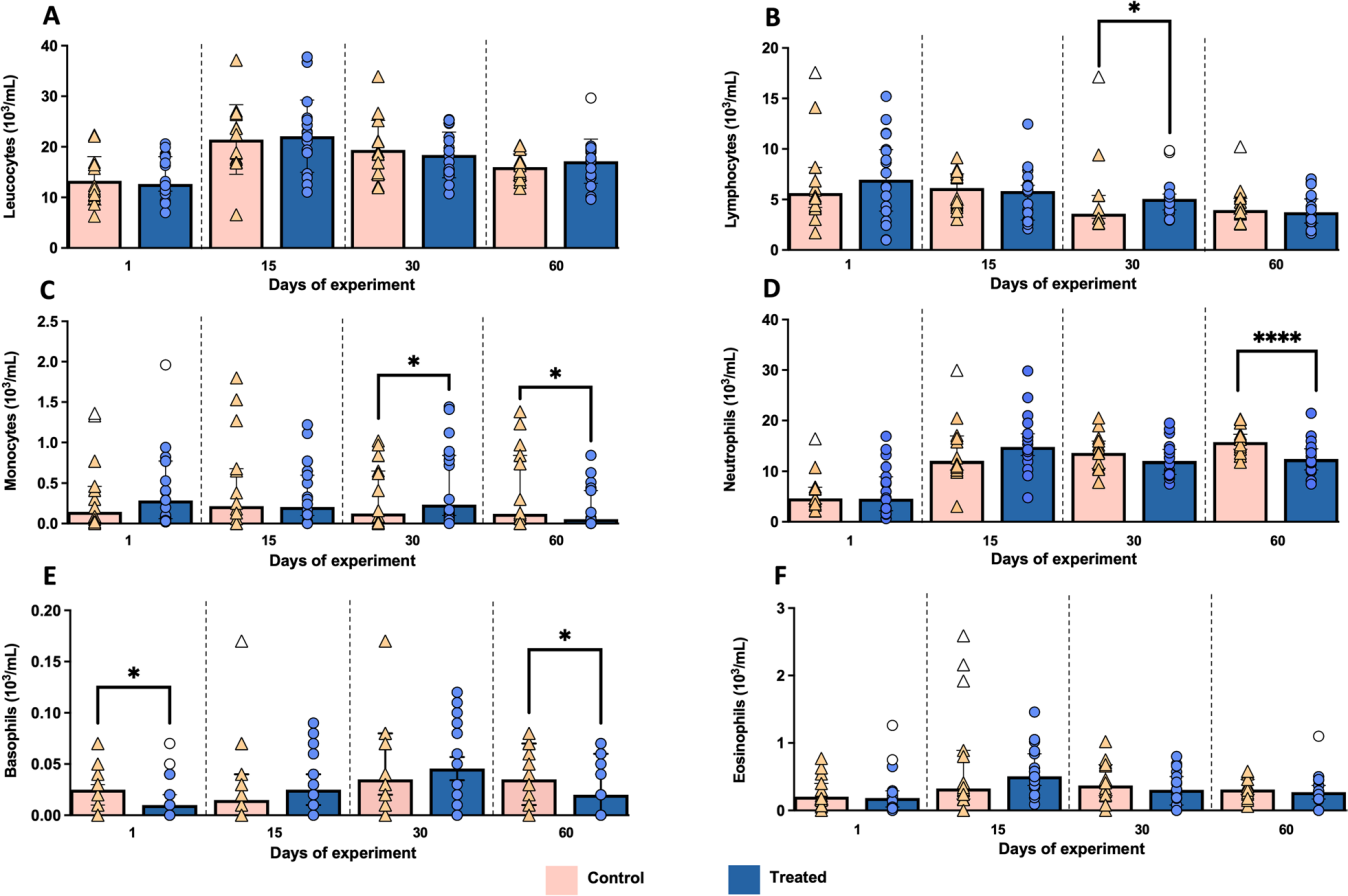
Graphical representations of leucocytes and the differential white blood cell count along the study. **A** Leucocytes. **B** Lymphocytes. **C** Monocytes. **D** Neutrophils. **E** Basophils. **F** Eosinophils. *P* < 0.05 (*) and *P* < 0.0001 (****). Bars represent the median of each group at different time points. Orange triangles (control group n = 14) and blue circles (treated group n = 18) represent the value of each animal within each group. Empty symbols represent outliers

Lymphocytes count displayed a significant increase in the treated group compared to the control one at day 30 of the study (*P* = 0.023) (Fig. 3B). This difference was also observed in the number of monocytes at the same time point (*P* = 0.013), which then showed a significant decrease at the end of the study (day 60) (*P* = 0.035) (Fig. 3C). With respect to neutrophils counting, a significant decrease was evidenced in the treated group at the end of the study (day 60) (*P* < 0.0001) (Fig. 3D). In addition, a significant decrease was observed in the number of basophils in the treated group on days 1 (*P* = 0.048) and 60 (*P* = 0.012) of the study in comparison with the control group (Fig. 3E). No significant changes were observed in the number of eosinophils during the whole study (Fig. 3F). A statistically significant Time × Treatment interaction was observed for both lymphocyte and neutrophil counts (*P* = 0.020 and *P* = 0.013, respectively; two factors ANOVA).

### Vitamin D_3_ and its metabolites analysis

**Fig. 4:**
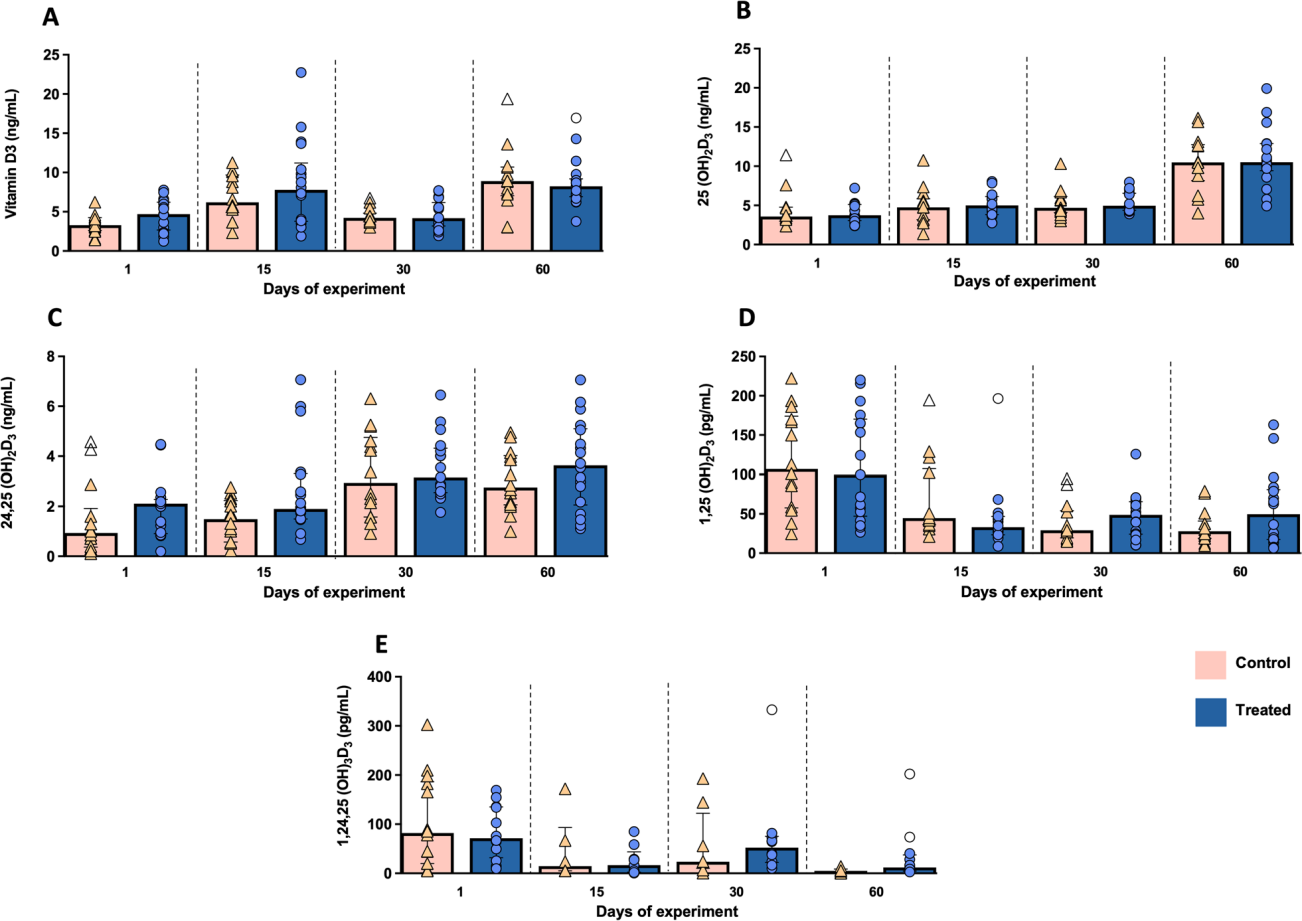
Graphical representations of the serum levels of vitamin D_3_ and its metabolites along the study. **A** Vitamin D_3_. **B** 25(OH)D_3_. **C** 1,25(OH)_2_D_3_. **D** 24,25(OH)_2_D_3_. **E** 1,24,25(OH)_3_D_3_ in both experimental groups. Bars represent the median of each group at different time points. Orange triangles (control group n = 14) and blue circles (treated group n = 18) represent the value of each animal within each group, whereas clear symbols represent outliers

Discrete but not significant changes were detected in vitamin D_3_ and its metabolites between experimental groups, due to wide individual variability (Fig. 4). Active vitamin D_3_ levels showed a similar trend in both experimental groups displaying a progressive although irregular increase throughout the study (Fig. 4A). Levels of 25(OH)_2_D_3_ remained plateau at the beginning of the study, presenting a marked increase in both groups at day 60 (*P* = 0.612) (Fig. 4B). A progressive increase was also observed in 24,25(OH)_2_D_3_ metabolite, which trended to be slightly higher in the treated group at the end of the study (*P* = 0.222) (Fig. 4C). The opposite trend occurred for 1,25(OH)_2_D_3_ and 1,24,25(OH)_3_D_3_ metabolites, which displayed a decrease in their serum levels along the study; however, higher values of both metabolites were evidenced in supplemented animals in comparison with the control group at days 30 (*P* = 0.372 and *P* = 0.359, respectively) and 60 (*P* = 0.111 and *P* = 0.083, respectively) of the experiment (Fig. 4D and E).

### IgA and IgG antibodies analysis

**Fig. 5:**
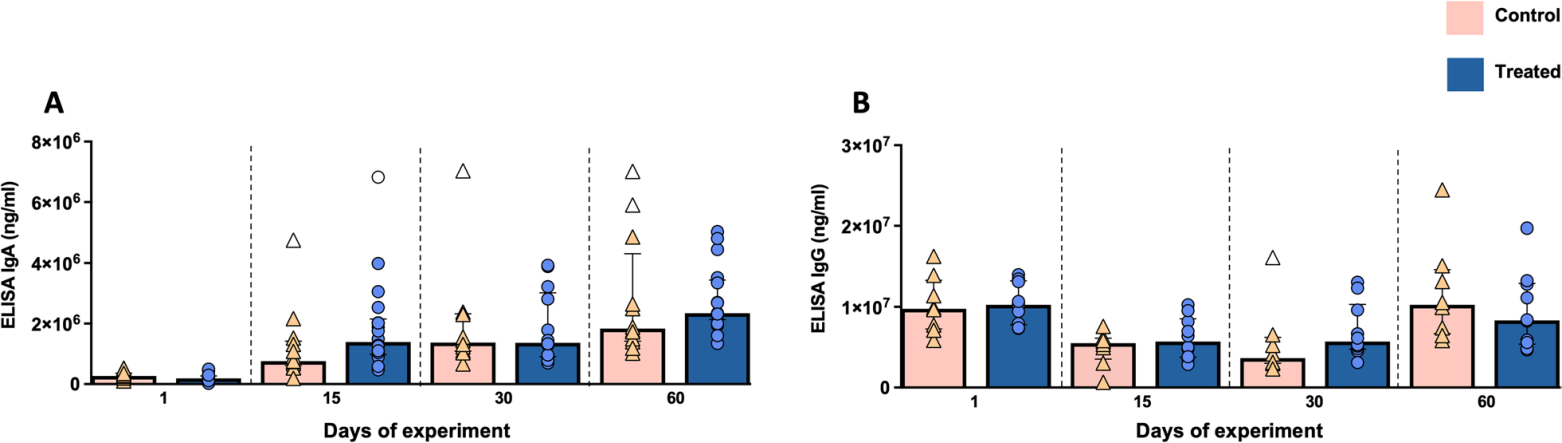
Graphical representations of IgA (**A**) and IgG (**B**) serum levels along the study. Bars represent the median of each group at different time points. Orange triangles (control group n = 14) and blue circles (treated group n = 18) represent the value of each animal within each group. Empty symbols represent outliers

A progressive increase in IgA serum levels was observed from day 1 of the study onwards, displaying slightly higher levels, although without statistically significant differences between groups along the study (*P* > 0.05), in supplemented pigs (Fig. 5A). On the other hand, IgG serum levels decreased in both experimental groups at days 15 and 30 but maintaining higher values in supplemented animals and recovering initial values at the end of the study period, although no significant differences were observed either (*P* = 0.295 and *P* = 0.146, respectively) (Fig. 5B). However, for IgG, statistical significance was observed in the Time x Treatment interaction (*P* = 0.026).

### Histological evaluation and morphometric analysis

All animals from control and treated groups were healthy along the study and no changes were evidenced at the histological evaluation. In general, all animals presented a mild infiltrate of mononuclear cells (lymphocytes and plasma cells) in the lamina propria of the small intestine, without evident signs of enteritis. Nonetheless, scattered animals from both experimental groups showed a moderate increase in the inflammatory infiltrate in the small intestine (duodenum, jejunum, and ileum), in addition to edema in the lamina propria.

**Fig. 6:**
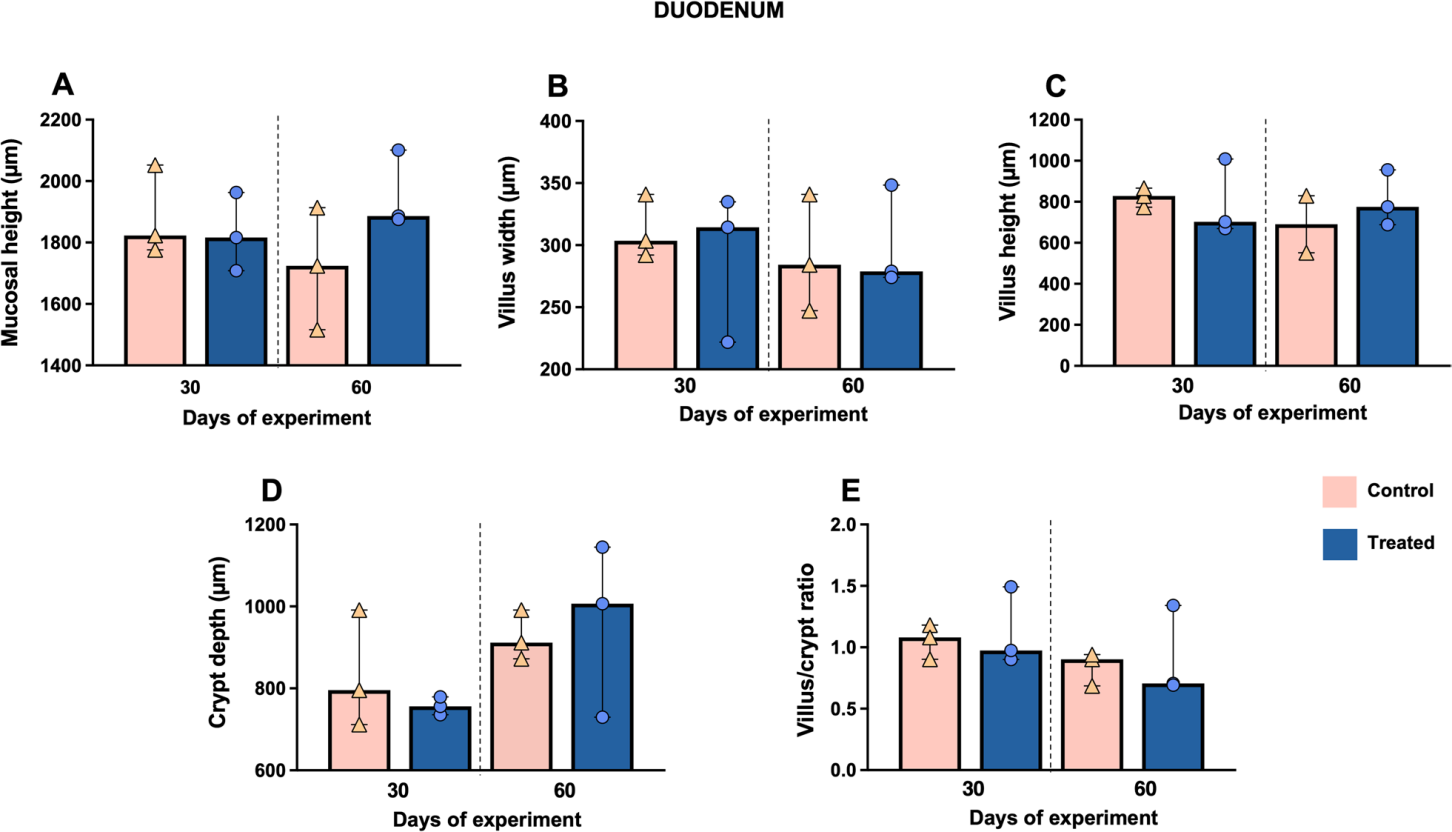
Morphometric analysis of the duodenum at days 30 and 60 of the study. Graphs show different measures including mucosal height (**A**), villus width (**B**), villus height (**C**), crypt depth (**D**) and villus/crypt ratio (**E**) in this intestinal segment. Bars represent the median of each group at different time points. Orange triangles (control group n = 3) and blue circles (treated group n = 3) represent the value of each animal within each group

Regarding the morphometric analysis, the mucosal height of the duodenum in the treated group at day 60 of the experimental study was higher compared to the control group (*P* = 0.400) (Fig. 6A), as well as an increase in the crypt depth parameter in both experimental groups from day 30 to day 60 of the study (Fig. 6D). No significant changes were observed between groups in villus width, villus height and villus/crypt ratio (*P* > 0.05) (Fig. 6B, C and E).

**Fig. 7:**
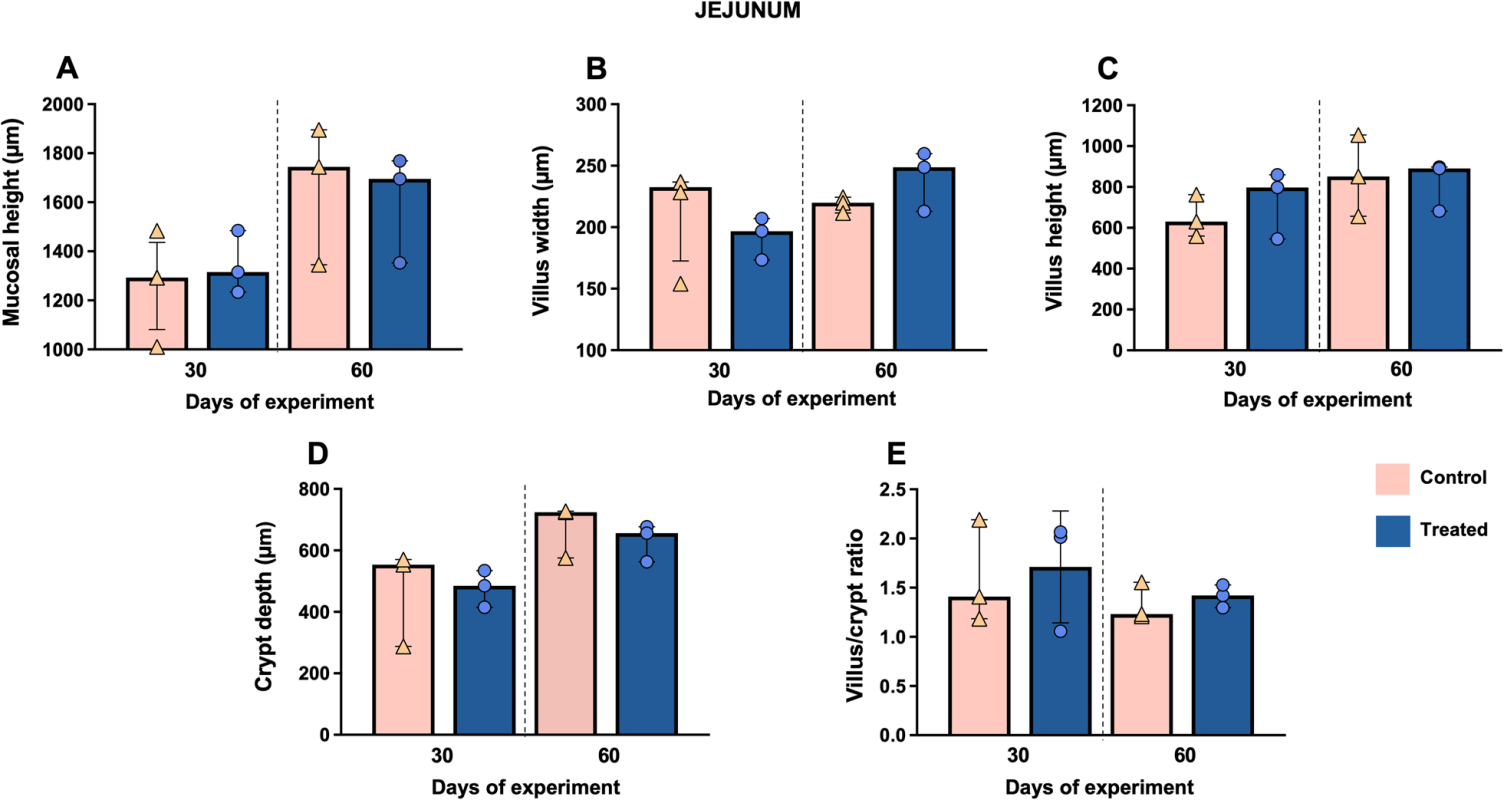
Morphometric analysis of the jejunum at days 30 and 60 of the study. Graphs show different measures including mucosal height (**A**), villus width (**B**), villus height (**C**), crypt depth (**D**) and villus/crypt ratio (**E**) in this intestinal segment. Bars represent the median of each group at different time points. Orange triangles (control group n = 3) and blue circles (treated group n = 3) represent the value of each animal within each group

In the jejunum, mucosal height, villus height and crypt depth parameters displayed a gradual increase from day 30 to 60 in both experimental groups (Fig. 7A, C and D). Moreover, higher values were observed in the treated group for villus width parameter at day 60 of the study (Fig. 7B). This is consistent with the existence of statistical significance (*P* = 0.036) when doing the bootstrap method. In addition to this, the villus/crypt ratio showed an increase in the treated group, mainly at day 30 of the experiment (median 2.01, IQR = 1.01) compared to the control group (median 1.41, IQR = 1.01), although without significant differences (*P* > 0.999) (Fig. 7E).

**Fig. 8:**
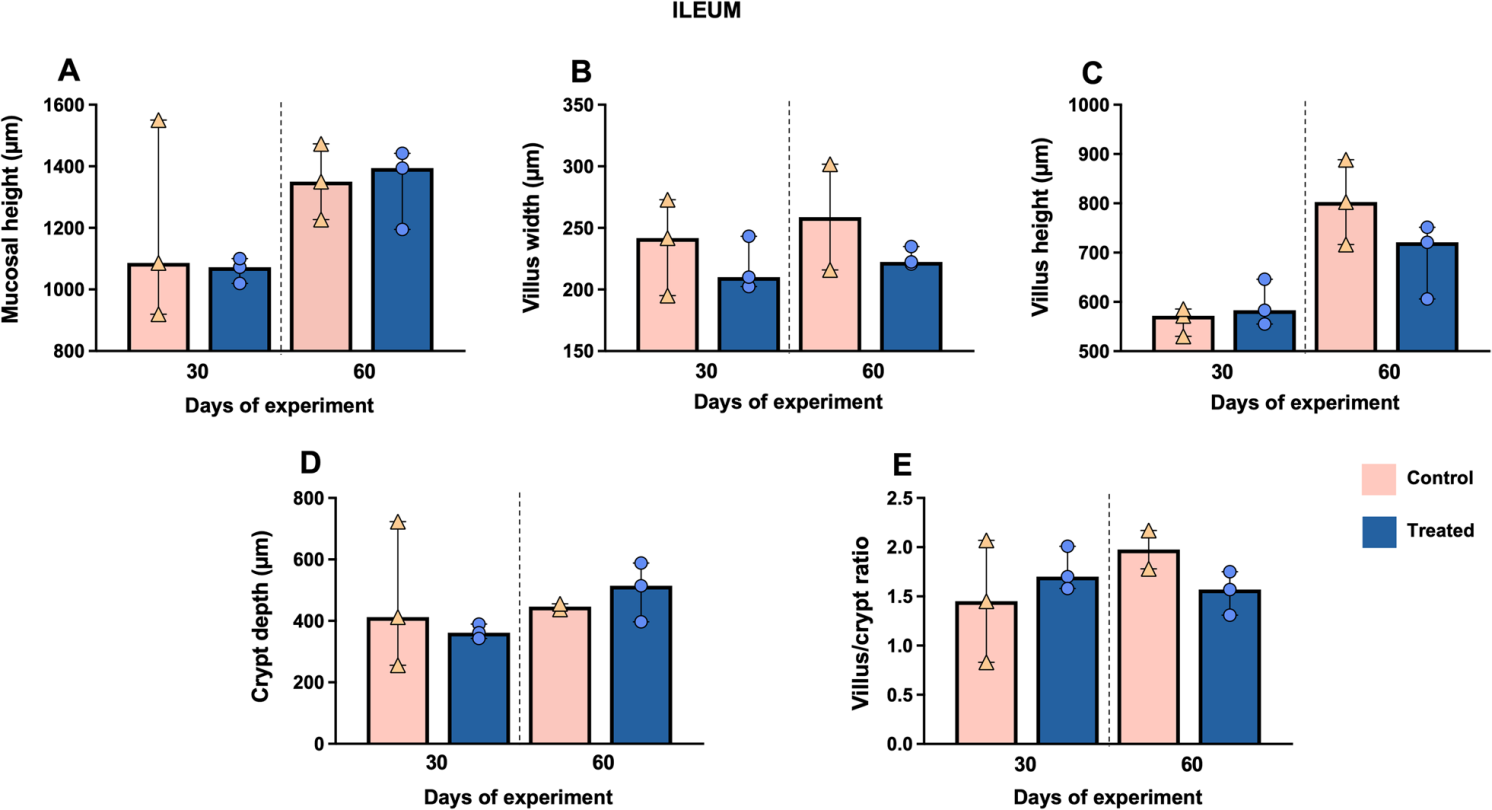
Morphometric analysis of the ileum at days 30 and 60 of the study. Graphs show different measures including mucosal height (**A**), villus width (**B**), villus height (**C**), crypt depth (**D**) and villus/crypt ratio (**E**) in this intestinal segment. Bars represent the median of each group at different time points. Orange triangles (control group n = 3) and blue circles (treated group n = 3) represent the value of each animal within each group

Regarding the ileum, Fig. 8A shows an increase in mucosal height in both experimental groups at day 60 of the study and in the crypt depth only in the treated group at the same time point (Fig. 8D). However, villus height, villus width and villus/crypt ratio showed an increase in the control group, compared to the treated group, at the end of the study (day 60) (*P* > 0.05) (Fig. 8B, C and E).

### Immunohistochemical analysis

#### IgA immunolabelled cells assessment

**Fig. 9:**
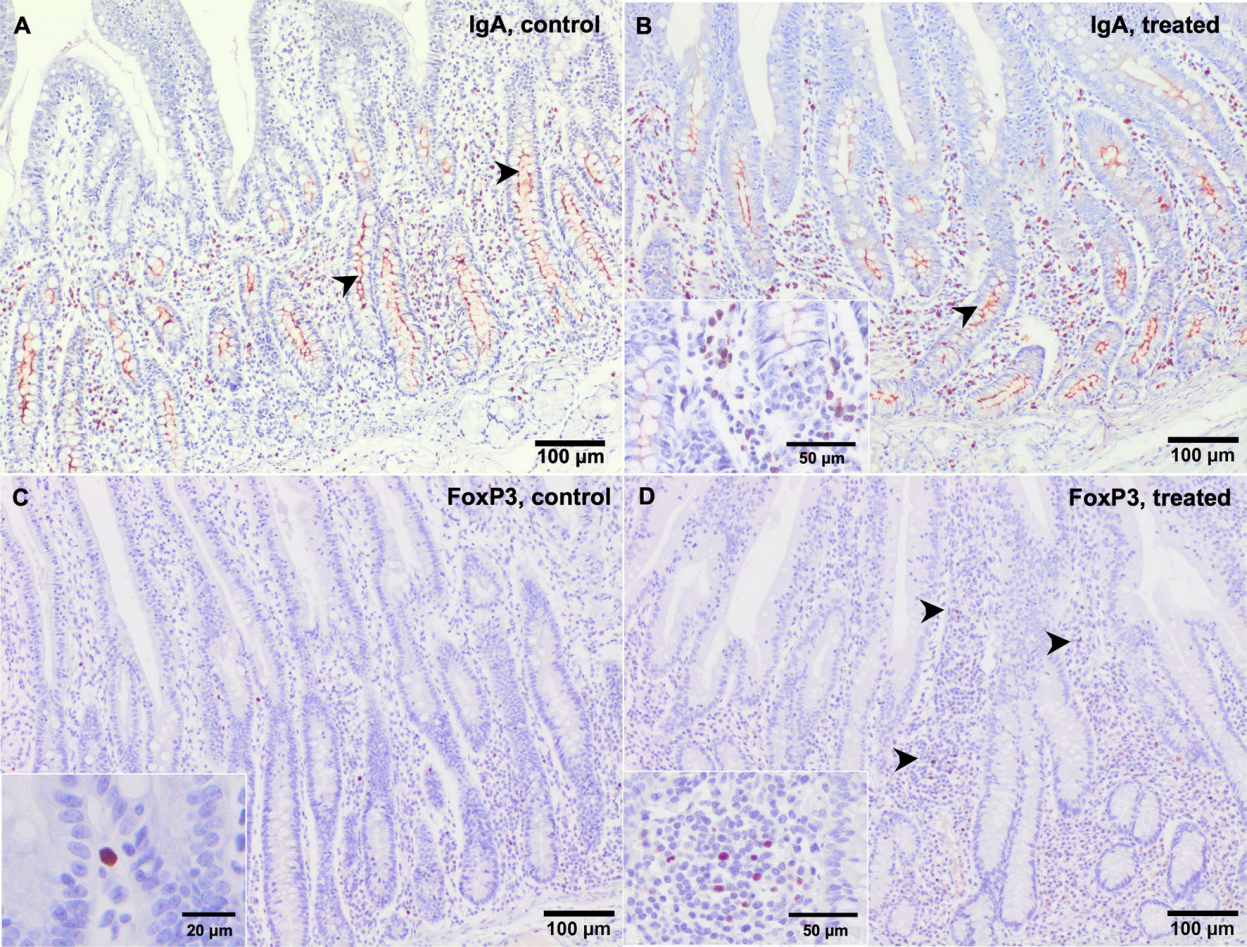
Immunolabelling against IgA and FoxP3 in the duodenum of animals euthanised at day 60. IgA expression in the lamina propria of the duodenum from a control animal (**A**) and from a treated animal (**B**). Arrowheads show IgA staining in the lumen of the crypts. Inset shows a detail of immunostained plama-like cells against IgA. FoxP3 expression in lymphocyte-like cells in the lamina propria of the duodenum from a control animal (**C**) and from a treated animal (**D**). Arrowheads show FoxP3^+^ cells in the lamina propria of the villi and upper half of the lamina propria of crypts. Inset shows the nuclear labelling in lymphocyte-like cells

**Fig. 10:**
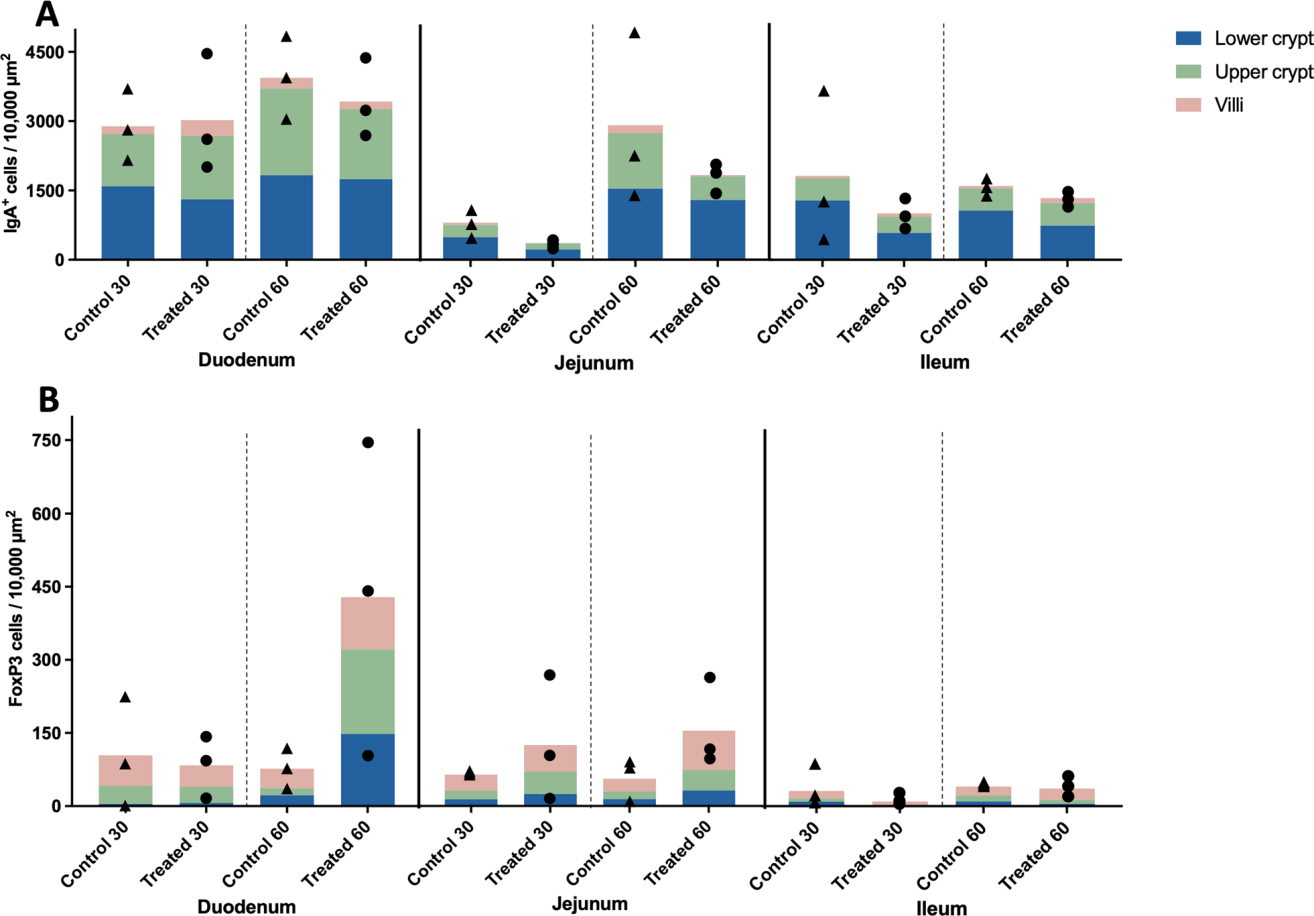
IgA (**A**) and FoxP3 (**B**) immunolabelled cells at days 30 and 60 of the study. The results are represented by the mean value at the level of villi, and the upper and lower half of the lamina propria of crypts. Black triangles and black circles represent individual values for each animal in the control group (n = 3) and treated group (n = 3), respectively, in each intestinal segment (duodenum, jejunum and ileum)

IgA staining was identified in the cytoplasm of plasma-like cells (Fig. 9B, inset) and in the luminal side of the crypt epithelium (Fig. 9A and B, arrowheads), mainly in crypts areas (upper and lower halves of the lamina propria of the crypts) and in a lesser extent in the lamina propria of the villi (Fig. 9A and B). Expression of IgA was mostly observed in the duodenum in both experimental groups and in a lesser extent in the jejunum and ileum (Fig. 10A). Moreover, a higher number of IgA^+^ cells was detected in the upper and lower halves of the lamina propria of the crypts in the three segments from the small intestine (Fig. 10A). Nevertheless, no differences were observed between the two experimental groups.

#### FoxP3 labelling

FoxP3 yielded a nuclear labelling in lymphocyte-like cells (Fig. 9C, inset), mainly located in the lamina propria of villi and the upper half of crypts in all the intestinal segments (duodenum, jejunum, and ileum) (Fig. 9D, arrowheads; Fig. 10B). A striking increase was observed in the number of FoxP3^+^ cells in the treated group in comparison with control group at day 60 of the study, increasing its expression mainly in the upper half of the lamina propria of crypts in the three intestinal segments (Fig. 10B); however, due to large interindividual variability no significant differences were observed between both groups.

## Discussion

Given the current restrictions on the use of pharmacological products, there is increasing pressure in identifying new substances, such as probiotics, prebiotics, enzymes, essential oils, and plant extracts or their combinations to cover the needs in animal feeding [18, 19]. Thus, dietary supplementation could become an alternative tool to maintain profitability, health status and animal performance in the pig industry. Vitamin D supplementation in weaned Iberian piglets resulted in our study in an improved growth performance and homogeneity of animals’ batches, as well as a higher frequency of Tregs in the gut mucosa.

Vitamin D is an essential micronutrient in animal diets, commonly known for its role in the regulation of calcium and phosphorus metabolism. However, the benefits of vitamin D go beyond that, being also involved in growth performance and immunity [2]. In this sense, several studies have assessed the impact of vitamin D supplementation on porcine health, focused mainly on gestating sows, farrowing and weaning piglets [9–12, 20–25]. However, the effect of vitamin D supplementation in later stages of pig production and under outdoor systems has been scarcely evaluated. Therefore, our study investigated the impact of supplementation with 1,25(OH)_2_D_3_ metabolite, the active form of vitamin D, on growth performance and on immunological and intestinal parameters for a period of two months postweaning in outdoor pigs. Control animals were fed with a basal diet containing vitamin D, whereas treated animals were fed with the same basal diet with an additional source of vitamin D, a product based on *Solanum glaucophyllum* extracts rich in 1,25(OH)_2_D_3_. It is noteworthy to highlight that the basal diet of animals during this production stage already contained a high level of supplementation with the native form of vitamin D and that all animals in the experiment had free access to outdoor from the middle stage of the experiment onwards, being able to synthesize vitamin D_3_ thanks to sunlight exposure. These aspects, together with specific features of the Iberian breed, may have impacted in the results obtained in both experimental groups, not only in the treated one, and they could have hamper obtaining significant results in some evaluated parameters when comparing both experimental groups, something that should be taken into consideration.

In our study, the impact of vitamin D_3_ supplementation on body weight, ADG, FCR and CV-BW parameters was evaluated. Previous studies also suggest a positive effect on these growth parameters in the progeny and in piglets’ body weights of sows supplemented with 25(OH)D_3_ [2, 24, 25]. It has been suggested that improvement on growth performance might be partly due to the activation of the growth hormone (GH)/insuline-like growth factor (IGF)-1 axis, which promotes bone, skeletal muscle, and general body growth [2]. However, how vitamin D interacts with GH/IGF-1 system has not been clarified yet [2, 26]. Interestingly, our results showed a greater body weight in the treated group along the study period, a parameter also affected by the Time x Treatment interaction, and a lower CV-BW in the supplemented group compared to the control one, mainly at the end of the study (day 60), which would help to obtain a greater homogeneity within batches.

Although no significant differences between both groups were found in our study, the supplementation of vitamin D on treated animals was associated with an increase of 1,25(OH)_2_D_3_ and 1,24,25(OH)_3_D_3_ values on serum. During the metabolism of vitamin D, 1,25(OH)_2_D_3_ is hydroxylated to form 1,24,25(OH)_3_D_3_ in target tissues [6], which could explain the parallel kinetics of both metabolites along the study.

Vitamin D plays also a role as an anti-infection and anti-inflammatory molecule, as has been previously demonstrated in commercial [9–11] and wild pigs [17]. In this sense, a positive effect in intestinal health was observed in supplemented piglets after rotavirus infection [9, 11]. In the study by Zhao et al*.* [9], dietary supplementation with high dose of vitamin D mitigated the effect of rotavirus infection in villus height compared to the supplementation with low dose of vitamin D. It has been demonstrated that 1,25(OH)_2_D_3_ has a protective role in the mucosal barrier due to the upregulation of tight junction and adherent junction protein expression [27–29], which would be related to an increased integrity of the intestinal mucosa and thus to improved gut health [29]. Due to the high individual variability and the low number of animals available in our study, only mild differences in the histomorphometry analysis of different intestinal sections were observed between the treated and the control groups in our study, however, the trends observed in our results call for further studies including larger number of animals.

The modulation of the immune response by vitamin D_3_ and its active metabolite 1,25(OH)_2_D_3_ has been associated with the expression of vitamin D receptor (VDR) on numerous cells of the immune system (monocytes, macrophages, natural killer cells, dendritic cells, T and B lymphocytes) [3, 5, 10]. Blood leukocytes are key activators and regulators of inflammation and homeostasis in swine [3]. In our study a mild increase in the number of lymphocytes and monocytes was observed in the treated group at day 30 of the experiment, as has been previously observed in weaning pigs supplemented with 25(OH)D_3_ [10]. Although our study was not performed under challenging conditions and further studies need to be conducted on this topic, the decrease in the number of neutrophils and monocytes together with the increase in the frequency of FoxP3^+^ cells point out to a modulation of the immune response in 1,25(OH)_2_D_3_ in the supplemented pigs. Furthermore, the significant Time x Treatment interaction observed for lymphocyte and neutrophil counts, highlight the dependence of the impact of the supplementation on the time of exposure to the supplementation and encourage evaluating the effect of this supplementation for longer periods.

FoxP3 is a nuclear transcription factor which plays a role in the differentiation of Tregs, a subset of CD4 T cells which act suppressing the immune response [30]. It has been described that 1,25(OH)_2_D_3_ could result in an enhancement of Tregs in human in vitro experiments [31] but its effect in swine has not been studied until the moment. Although no statistically significant differences were detected in our study, a striking increase in the number of FoxP3^+^ cells was observed in the intestinal tract of treated animals at the end of the study, which point out to an immunomodulatory effect of this molecule in the intestinal mucosa. Concerning the location, the number of positive cells was greater in the lamina propria of the crypts compared to villus, being mainly expressed in duodenum and decreasing progressively in jejunum and ileum. This progressive decrease could be related to the reduction of amount of food antigens which are destroyed along the small intestine, as was suggested by Junginger et al*.* [32].

Vitamin D_3_ and 1,25(OH)_2_D_3_ can also modulate humoral immune response [7, 33]. IgA plays a pivotal role protecting mucosal surfaces from virus, bacteria, and toxins by preventing the binding to the mucosal surface or through direct neutralization [34]. In our study supplemented pigs displayed slightly higher levels of IgA in serum compared to control animals, although this trend was not confirmed at intestinal level. Immunohistochemical assessment of IgA expression in the small intestine revealed higher number of IgA^+^ cells in the lamina propria of crypts, as was previously reported by other authors [35], however, no differences between treated and control animals were detected in our study. On the other hand, IgG is the predominant isotype found in the body and its function is related to the neutralization of toxins and viruses [34]. An increase in IgG serum levels has been already reported by other authors in weanling piglets fed with 25(OH)D_3_ [24], however, in our study this change was not evident, although its serum concentration was affected by the Time x Treatment interaction. Our results indicate a mild positive effect of vitamin D on humoral immunity stimulation, mostly in IgA serum levels, in the conditions of the present study. Longer supplementation with 1,25(OH)_2_D_3_ might result in a more apparent effect at this level.

According to our results, one of the main limitations of this study is the low sample size along the study due to management and operability constraints of the farm where the experimental study was carried out; however, the housing and management for all piglets were identical, thereby allowing a valid comparison of both experimental groups.

## Conclusions

Our results show that supplementation of Iberian piglets for two months postweaning with 1,25(OH)_2_D_3_ in an indoor/outdoor system resulted in an improvement of growth performance, together with greater homogeneity of animals in the treated group compared to control one. These findings were accompanied by an increase of IgA in the serum and FoxP3^+^ cells in the small intestine of the supplemented animals. However, the low number of animals monitored together with the individual variability call for further studies focused on confirming the effect of this molecule during longer supplementation periods as well as on later stages of the pig production cycle.

## Methods

### Animals and experimental design

The present experiment conducted to investigate the benefits of supplementing the diet of piglets at postweaning with vitamin D_3_ metabolites, was carried out following the European Union guidelines (Directive 2010/63/EU) and was approved by the ethics committee of the Junta de Andalucía (Approval number: 01/25/2022/005). Briefly, a total of 252 purebred Iberian piglets of 4 weeks of age, both sexes and not castrated were blocked by sex and weight and divided into two experimental groups (control group *vs* treated group) at the farm “Las Rozuelas del Valle” (Torrecampo, Cordoba, Spain), with an equal number of both sexes in each group. Each group was formed by 6 batches of approximately 21 animals. The animals were housed in these pens during the first 30 days postweaning with control of environmental conditions such as temperature and ventilation to avoid temperatures over 26 degrees. Then, they were grouped together according to their treatment within a single batch per treatment, with a free access to outdoor, but with no access to natural feed resources, such as grass, acorns, or other type of food. Animals from control group were fed with a standard commercial feed in form of flour, which already included vitamin D (containing 2000 IU in the starter and 1000 IU in the adaptation diets, respectively), and the treated group received the same standard commercial feed together with a supplementation in powder form of a product based on plant extracts rich in the dihydroxylated form of vitamin D_3_ (1,25(OH)_2_D_3_) (Panbonis®, Andersen, Barcelona, Spain; 100 ppm; 10 μg of 1,25(OH)_2_D_3_ glycoside/g of product). Both food and water were provided ad libitum. Tables 2 and 3 represent the diet and nutrient composition for both experimental groups along the study, respectively. Thirty-six out of the total of pigs were monitored throughout the study (control group n = 18; treated group n = 18). However, 4 control animals displayed growth retard along the study and were individually housed in an apart pen for management issues; thus, these animals were removed from the analyses to keep only those animals which completed the whole period of the experiment in their corresponding batches. The mean body weight of the control and treated groups at the beginning of the study was 5.0 ± 1.23 kg and 5.1 ± 1.09 kg, respectively (4 weeks old, weaned piglets). At 1, 15, 30 and 60 days postweaning, the individual body weight and feed intake from each batch of monitored animals were recorded and the ADG, the FCR as well as the CV-BW of the weights between both experimental groups were calculated. Blood and sera samples were collected by venipuncture from jugular vein at these time points from the 32 animals that finally took part in the experimental study (control group n = 14; treated group n = 18) for further investigations. Additionally, clinical signs and abnormal behavior were daily monitored in these animals. Furthermore, 3 animals from each experimental group, out of the total of 252 animals, and different from those selected for blood sampling, were humanely euthanized at days 30 and 60 of the experimental study, and samples from different sections of the small intestine (duodenum, jejunum, and ileum) were collected and fixed in 10% neutral-buffered formalin for histopathological and morphometry analyses.

**Table 2:** Diet composition for both experimental groups along the study

Ingredients	Starter^1^	Adaptation^2^
Barley grain	31.6	38.5
Corn grain	24	36.4
Wheat grain	17	–
Pre-mix	10^3^	5^4^
Soybeen meal, 47% CP^5^	9.2	17.3
Reprofish alter micrum	2.5	–
Lard	2.2	1.5
Sugar beet pulp	1.5	–
Sepiolite	1	–
Calcium carbonate	–	0.7
Organic acids	0.6	0.6
Salts of fatty acids	0.4	–

^1^Starter diet was administered to both experimental groups during the first 30 days of the study. ^2^Adaptation diet was administered to both experimental groups from the day 30 of the study onwards. ^3^Pre-mix containing 2000 IU of vitamin D. ^4^Pre-mix containing 1000 IU of vitamin D. ^5^CP: Crude Protein

**Table 3:** Nutrient composition of the diet for both experimental groups along the study

Nutrient composition	Starter^1^	Adaptation^2^
Volume (kg)	1	1
Dry matter (%)	88.91	88.76
Crude protein (%)	15.96	16.49
Crude fat (%)	4.52	3.94
Crude fiber (%)	3.35	3.71
Starch (%)	41.57	42.76
Starch + sugar (%)	45.67	46.29
Calcium (%)	0.64	0.7
Phosphorus (%)	0.48	0.43
Lysine (%)	1.07	0.95
Methionine (%)	0.38	0.31
Methionine + cystine (%)	0.67	0.62
Threonine (%)	0.7	0.67
Tryptophan (%)	0.21	0.21
ADF (%)^3^	3.97	4.45
NDF (%)^4^	11.18	11.62
Ash (%)	5.77	4.68
Chlorine (%)	0.13	0.07
Sodium (%)	0.08	0.02
Metabolizable energy (Kcal/kg)	2450.15	2440.44

^1^Starter diet was administered to both experimental groups during the first 30 days of the study. ^2^Adaptation diet was administered to both experimental groups from the day 30 of the study onwards. ^3^ADF: Acid Detergent Fiber. ^4^NDF: Neutral Detergent Fiber

### Blood and sera analysis

A differential white blood cell count (lymphocytes, monocytes, neutrophils, eosinophils, and basophils) was performed with whole blood samples. Serum samples were used for the determination of vitamin D_3_ and its metabolites (25(OH)D_3_; 1,25(OH)_2_D_3_; 24,25(OH)_2_D_3_; 1,24,25(OH)_3_D_3_) using a method based on an automatic solid-phase extraction unit on-line connected to a liquid-chromatograph tandem mass spectrometer arrangement (SPE-LC–MS/MS) [13]. The method was previously validated by applying the Vitamin D Standardization Program (VDSP) and according to external quality assurance scheme (DEQAS). Calibration curves for quantification were obtained through the ratio between the chromatographic peak area of each analyte and the corresponding deuterated standard. SPE-LC–MS/MS analysis and sample preparation have been described elsewhere (see [36] for more detail).

Additionally, serum IgA and IgG concentrations were determined using enzyme linked immunosorbent assay commercial kits [pig IgA ELISA kit and pig IgG ELISA kit, respectively (Bethyl Laboratories, Texas, USA)], following the manufacturer’s instructions. Samples were diluted 1:7200 for the analysis of IgA and 1:388,800 for IgG. Results were expressed in ng/mL and the minimum detectable concentration were 25.4 μg/mL for IgA and 647.6 μg/mL for IgG.

### Histopathological evaluation and histomorphometric analyses

Four-micrometer tissue sections from duodenum, jejunum and ileum were stained with hematoxylin and eosin and blindly examined by two pathologists for the histopathological evaluation. The presence of inflammatory cells and edema in lamina propria were evaluated in each section of small intestine. For histomorphometry, sections were submitted to analysis by using the software ImageJ (version 1.52a Wayne Rasband National Institutes of Health, USA). Different measures including mucosal height, villus width at 2 points, villus height on both sides, crypt depth and villus/crypt ratio were calculated in 10 villi of each intestinal section at 10× magnification. Results were expressed in μm.

### Immunohistochemical analysis

The Avidin–Biotin–Peroxidase Complex technique (ABC Vector Elite, Vector Laboratories, Burlingame, CA, USA) was performed to detect the expression of IgA (polyclonal goat anti-pig IgA, Bethyl Laboratories) and FoxP3 (clone FJK-16s, eBioscience, Barcelona, Spain) in the samples from small intestine. In brief, 4 μm tissue sections from each sample were dewaxed and rehydrated in xylene and descending grades of alcohol, respectively, followed by endogenous peroxidase inhibition using 3% of H_2_O_2_ in methanol for 30 min in darkness. An enzymatic digestion (protease form *Bacillus licheniformis*, Sigma-Aldrich, USA) and a heat pretreatment (citrate pH 6.0 in autoclave, 10 min at 121 °C) were carried out for IgA and FoxP3, respectively. After phosphate-buffered saline (PBS) washes, incubation with 100 μl of 2% bovine serum albumin (BSA) was assessed for IgA. In the case of FoxP3, washes were performed with tris buffered saline with 0.2% of tween 20 (TBST 20) and 10% normal goat serum (NGS) was applied as blocking solution. IgA and FoxP3 primary antibodies were applied and incubated overnight at 4 °C in a humidity chamber diluted 1:3,000 and 1:100, respectively. For negative controls, the primary antibody was replaced by BSA or NGS in each case to confirm the lack of non-specific biding. After incubation, the slides were again incubated with the corresponding biotinylated secondary antibody for 30 min at room temperature in darkness. Then, the Avidin–Biotin–Peroxidase Complex was applied for 1 h under the same conditions. Labelling was visualized with the Vector NovaRed substrate kit (Vector Laboratories). Finally, slides were counter-stained with Harris hematoxylin, to later be dehydrated in ascending grades of alcohol and mounted with Eukitt® (Orsatec GmbH, Bobingen, Germany).

Immunolabeled cells were identified and automatically counted by using the QuPath software (version 0.3.2) [37], analyzing 5 randomly chosen fields at 10× magnification of each section of the small intestine. In each field, the number of positive cells were determined in 3 different areas of lamina propria: the villus, the upper half of the lamina propria with crypts and the lower half of the lamina propria with crypts. Results were expressed as the number of positive cells per 10,000 μm^2^ of tissue [38].

### Statistical analysis

Statistical differences for each parameter analyzed at the different time points (1, 15, 30 and 60 days) of the experiment between control and treated groups were evaluated for approximate normality of distribution using the D’Agostino and Pearson omnibus normality test, followed by the non-parametric Kruskal–Wallis test for multiple comparisons and the Mann–Whitney non-parametric U test for unpaired groups (GraphPad Prism software version 9.2.0, Inc., San Diego, CA, USA). Additionally, analysis of variance was performed in the CV-BW to determine statistical differences by F test, and the existence of statistical significance in the Time x Treatment interaction was also assessed for each variable through a General Linear Model (GLM) (Statistical for Windows software version 8, USA). For those parameters coming from histomorphometric and immunohistochemical analysis, bootstrap was performed with the software IBM SPSS Statistics version 29.0.0.0 (241) (United Kingdom). A *P*-value lower than 0.05 (*), 0.01 (**), 0.001 (***), and 0.0001 (****) was considered statically significant.

## Data Availability

The datasets used and analyzed during the current study are available from the corresponding author on reasonable request.

## Abbreviations

ADG: Average daily gain
FCR: Feed conversion ratio
CV-BW: Coefficient of variation of body weight
Tregs: Regulatory T lymphocytes
IQR: Interquartile range
PBS: Phosphate-buffered saline
BSA: Bovine serum albumin
NGS: Normal goat serum
